# Protocol for analyzing the function of the type VI secretion system of the oral symbiont *Aggregatibacter aphrophilus* in targeting pathobionts

**DOI:** 10.1016/j.xpro.2024.103415

**Published:** 2024-10-25

**Authors:** Jan Oscarsson, Kai Bao, Akiko Shiratsuchi, Jonas Grossmann, Witold Wolski, Kyaw Min Aung, Mark Lindholm, Anders Johansson, Ferdousi Rahman Mowsumi, Sun Nyunt Wai, Georgios N. Belibasakis, Nagihan Bostanci

**Affiliations:** 1 Oral Microbiology, Department of Odontology, Umeå University, Umeå, Sweden; 2 Division of Oral Health and Periodontology, Department of Dental Medicine, Karolinska Institutet, Alfred Nobels Allé 8, 14104 Huddinge, Stockholm, Sweden; 3 Department of Liberal Arts and Sciences, Graduate School of Medicine, Sapporo Medical University, Sapporo, Hokkaido 060-8556, Japan; 4 Functional Genomics Center Zurich, ETH Zürich and University of Zürich, Zürich, Switzerland; 5 Swiss Institute of Bioinformatics (SIB) Quartier Sorge-Batiment Amphipole, 1015 Lausanne, Switzerland; 6 Department of Molecular Biology and the Umeå Centre for Microbial Research (UCMR), Umeå, Sweden; 7 Laboratory for Molecular Infection Medicine Sweden (MIMS), Umeå University, 90187 Umeå, Sweden

**Keywords:** genomics, microbiology, molecular biology

## Abstract

Here, we present a protocol for evaluating type VI secretion system (T6SS)-dependent fitness of the oral symbiont *A. aphrophilus* using biofilm competition assays and metaproteomics. We describe steps for designing T6SS-specific mutants. We then detail procedures for using them in competition assays with the pathobiont *A. actinomycetemcomitans* and in biofilm models, analyzing metaproteomes to assess the impact of the T6SS on multiple pathobionts. The biofilm model is designed to mimic the oral plaque ecosystem and includes seven species.

For complete details on the use and execution of this protocol, please refer to Oscarsson et al.^1^

## Highlights

• Design and test T6SS-specific mutants in biofilm and competition assays
• Perform dual-species competition assays with *A. actinomycetemcomitans* as prey
• Use a seven-species biofilm model mimicking the dental plaque ecosystem
• Analyze biofilm metaproteomes to assess T6SS impact on oral pathobionts

## Abstract (editor highlights)

Publisher’s note: Undertaking any experimental protocol requires adherence to local institutional guidelines for laboratory safety and ethics.

## Graphical abstract

**Figure:**
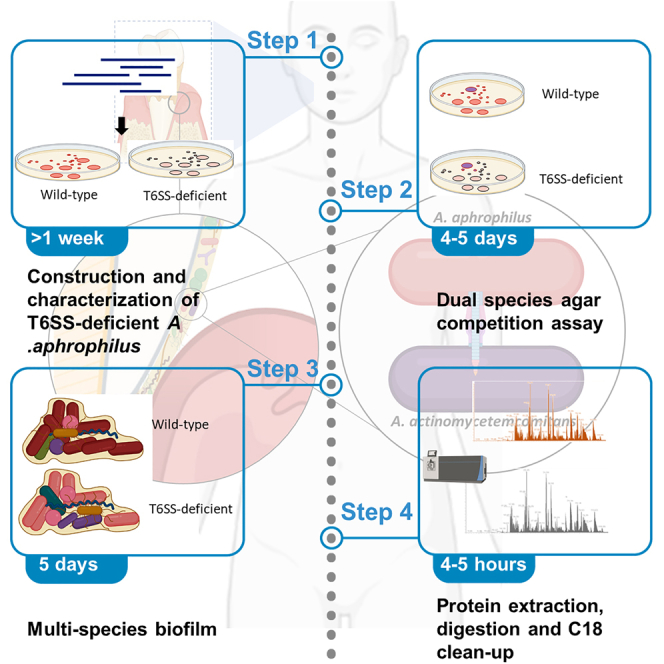

## Before you begin

The following protocols outline the precise procedures for PCR-based generation of type VI-secretion-specific mutants of *A. aphrophilus*, using dual-species agar competition assays, the multi-species oral biofilm model, and subsequent metaproteome analyses to assess the role of this nanomachinery in the fitness of this organism against pathobionts. In these procedures, the bacterial species and multispecies biofilms are kept in incubators at 37°C with 5% CO_2_. For transformation assays, prepare in advance, as described below, Trypticase soy broth (TSB), and TSB agar plates containing heat-inactivated horse serum (sTSB), with and without 100 μg/mL kanamycin (final concentration). For dual-species agar competition it is necessary to isolate spontaneous streptomycin- and rifampicin-resistant derivatives of the *A. aphrophilus* and *A. actinomycetemcomitans* model strains, respectively as detailed below, which are needed in these assays. Moreover, for multispecies biofilms, we recommended inoculating all needed strains from agar plate to broth 3 days (on the Friday before the experiments) ahead of the experiment.

***Note:*** If not stated otherwise, reagents can be substituted with similar alternatives from different vendors.

### Institutional permissions

All procedures were conducted in accordance with the guidelines of the local ethics committee at the Medical Faculty of Umeå University, which are in compliance with the Declaration of Helsinki (64th WMA General Assembly, Fortaleza, October 2013). No live vertebrates or higher invertebrates are involved in this work.

## Key resources table

**Table:** 

REAGENT or RESOURCE	SOURCE	IDENTIFIER
**Antibodies**		

Rabbit polyclonal antiserum specific for *V. cholerae* Hcp (used at 1:5,000 final concentration)	Ishikawa et al.^2^	RRID: AB_2313773
Anti-rabbit horseradish peroxidase – conjugate (used at 1:10,000 final concentration)	Jackson ImmunoResearch, Newmarket, UK	RRID: AB_2313773

**Bacterial and virus strains**		

*A. aphrophilus* strains HK83	Culture Collection University of Gothenburg (CCUG)	CCUG 49494
*A. aphrophilus* strains CCUG 11575	CCUG	CCUG 11575
*A. aphrophilus* strains NJ8700	CCUG	NJ8700
*A. aphrophilus* strains Aap-4K	Isolated from a patient^3^^,^^4^	Aap-4K
*A. aphrophilus* strains Aap-12K	Isolated from a patient^3^^,^^4^	Aap-12K
*A. aphrophilus* strains Aap-13K	Isolated from a patient^3^^,^^4^	Aap-13K
*A. aphrophilus* strains Aap-21K	Isolated from a patient^3^^,^^4^	Aap-21K
*A. aphrophilus* strains Aap-29K	Isolated from a patient^3^^,^^4^	Aap-29K
*A. aphrophilus* strains Aap-30K	Isolated from a patient^3^^,^^4^	Aap-30K
*A. aphrophilus* strains Aap-32K	Isolated from a patient^3^^,^^4^	Aap-32K
*A. aphrophilus* strains Aap-53K	Isolated from a patient^3^^,^^4^	Aap-53K
*A. aphrophilus* strains AHI-3151	Isolated from a patient^5^^,^^6^	AHI-3151
*A. aphrophilus* strains IH-90256	Isolated from a patient^5^^,^^6^	IH-90256
*A. aphrophilus* strains IH-90274	Isolated from a patient^5^^,^^6^	IH-90274
*A. actinomycetemcomitans* strain D7SS	Isolated from a patient^7^	D7SS
*A. actinomycetemcomitans* strain JP2	Bao et al.^8^	OMZ 295
*Actinomyces oris*	Bao et al.,^8^; Bostanci et al.^9^	OMZ 745
*Fusobacterium nucleatum* subsp. *nucleatum* KP-F2	Bao et al.^8^	OMZ 598
*Streptococcus oralis* SK248	Bostanci et al.^9^	OMZ 607
*Streptococcus mutans* UA159	Thurnheer et al.^10^	OMZ 918
*Veillonella dispar* ATCC 17748T	Bostanci et al.,^9^ Bao et al.^11^	OMZ 493

**Chemicals, peptides, and recombinant proteins**		

Tryptone	Difco	Cat#0123-01-1
Yeast extract	Difco	Cat#0127-01-7
Horse serum	Håtunalab AB	Product numbers 150, or 153 depending on the amount that is ordered (100 or 500 mL)
Blood agar plates	Laboratoriemedicin, Region Västerbotten, Technical Biochemical section	Product number TBK 14507
KNO_3_	Sigma-Aldrich	Cat#P6083
NaCl	Sigma-Aldrich	Cat#S7653
Glucose	Sigma-Aldrich	Cat#G7528
Cysteine. HCl	Sigma-Aldrich	Cat#30078
KH_2_PO_4_	Sigma-Aldrich	Cat#S7795
Na_2_HPO_4_.2 H_2_O	Sigma-Aldrich	Cat#P5655
Iodoacetamide	Sigma-Aldrich	Cat#71507
Urea	Sigma-Aldrich	Cat#I1149
Clarity western ECL substrate	Sigma-Aldrich	Cat#U0631
Tris/HCl	Sigma-Aldrich	Cat#T6666
triethylammonium bicarbonate	Thermo Fisher Scientific	Cat#90114
Trifluoroacetic acid	Sigma-Aldrich	Cat#1081780050
Trypsin	Promega	Cat#V511C
Methanol	Fisher Scientific	Cat#10031094
Formic acid	VWR	Cat#84865.180
Acetonitrile	VWR	Cat#14261

**Critical commercial assays**		

Ready-To-Go PCR beads	Cytiva	Cat#407513-STR
Microcon YM-30 centrifugal filter unit	Sigma-Aldrich	Cat#UFC503008
QIAquick PCR purification kit	QIAGEN	Cat# 28104
C18 disk core	Fisher Scientific	Cat#13110016

**Deposited data**		

Proteomic raw files	ProteomeXchange	PXD042723

**Oligonucleotides**		

The *A. actinomycetemcomitans*-specific primers 5′- CTAGGTATTGCGAAACAATTTG -3 (forward) and 5′- CCTGAAATTAAGCTGGTAATC -3′ (reverse)	Kirakodu et al.^12^	N/A
The *A. aphrophilus*-specific primers 5′-CCTACACCAGCGTTTATTTC-3′ (forward) and 5′-CTGAGGTTTACGCCAGTC -3′ (reverse)	Lindholm et al.^4^	N/A
Cyanine 3 -labeled *A. actinomycetemcomitans* 16S rRNA oligonucleotide probe Act639 5′-CTCCAGACCCCCAGTATG-3′	Thurnheer et al.^13^	Act639
The *hcp* gene replacement in *A. aphrophilus* upstream fragment forward primer *hcp*_F2 (5′-CGAGCGCAGGATTATAGCAGCT-3′)	This work	N/A
The *hcp* gene replacement of *hcp* in *A. aphrophilus* upstream fragment reverse primer *hcp*_R2 (5′- AAACGCTGGTGGATCCATAGAATTCTC-3′)	This work	N/A
The *hcp* gene replacement of in *A. aphrophilus* downstream fragment *hcp*_F3 (5′-GATGACTGGCGGATCCCTCAGGTT-3′)	This work	N/A
The gene replacement of *hcp* in *A. aphrophilus* downstream fragment *hcp*_R3 (5′-CACCGCTTGTGTATTGGCAGTGGC-3′)	This work	N/A
The kanamycin resistance cassette primer H7R (5′-GGACGGCGGCTTTGTTGAATAAATCG-3′),	This work	N/A
FAM-labeled *A. aphrophilus* 16S rRNA oligonucleotide 5′-CTCTAGACCCCCAGTCTG-3′	This work	Aaph639
pUC4K	Vieira et al.^14^	GenBank X06404; https://www.ncbi.nlm.nih.gov/genbank/

**Software and algorithms**		

Quantable packages (version 0.3.8)	N/A	https://github.com/protViz/quantable
Progenesis QI for proteomics (version 4.1 Nonlinear Dynamics)	Nonlinear Dynamics	https://www.nonlinear.com/progenesis/qi-for-proteomics/

**Other**		

*Candida albicans*	OMZ	OMZ 110

## Materials and equipment

We recommend preparing Solutions A and B the week before the experiment, while SNB (including SNB1 and SNB2) can be prepared a few weeks in advance and stored at 4°C. The final FUM/Sö./0.3%G medium should be prepared the afternoon before the experiment.

**Table:** FUM + 0.3% Glucose in Sörensen’s buffer (pH 7.2)

Reagent	Final concentration	Amount
Solution A	N/A	200 mL
Solution B	N/A	12.5 mL
SNB Solution	N/A	37.5 mL
**Total**	**N/A**	**250 mL**

Prepared the afternoon before the experiment. Store at 4°C until the end of the experiment.

***Note:*** Solution B and RTF solution are produced separately to avoid precipitation.

**Table:** Solution A

Reagent	Final concentration	Amount
Tryptone (Difco, 0123-01-1)	N/A	2.5 g
Yeast Extract (Difco, 0127-01-7)	N/A	1.25 g
KNO_3_	N/A	250 mg
NaCl	N/A	500 mg
Sörensen’s buffer	N/A	200 mL
**Total**	**N/A**	**200 mL**

Autoclave 20 min at 121°C.

Prepare a few weeks in advance and stored at 4°C up to 1 month.

**Table:** Solution B

Reagent	Final concentration	Amount
Glucose	N/A	750 mg
Cysteine. HCl	N/A	125 mg
Na_2_CO_3_	N/A	125 mg
Sörensen’s buffer	N/A	12.5 mL
**Total**	**N/A**	**12.5 mL**

Filter sterilization.

Prepare a few weeks in advance and stored at 4°C up to 1 month.

**Table:** Sörensen’s buffer, pH 7.2

Reagent	Final concentration	Amount
KH_2_PO_4_	9.078 g/L	330 mL
Na_2_HPO_4_.2 H_2_O	11.876 g/L	670 mL
**Total**	**N/A**	**1 L**

Store at 4°C for up to 1 year or until precipitates appear.

**Table:** Salt Nutrient Buffer (SNB)Solution

Reagent	Final concentration	Amount
SNB 1 stock solution	N/A	18.75 mL
SNB 2 stock solution	N/A	18.75 mL
**Total**	**N/A**	**37.5 mL**

Filter sterilization. Prepare just before use.

**Table:** SNB 1 stock solution

Reagent	Final concentration	Amount
K_2_HPO_4_	6 g/L	6 g
distilled H_2_O	N/A	1000 mL
**Total**	**N/A**	**1 L**

Store at 4°C for up to 1 year or until precipitates appear.

**Table:** SNB 2 stock solution

Reagent	Final concentration	Amount
NaCl	12 g/L	12 g
(NH_4_)_2_SO_4_	12 g/L	12 g
KH_2_PO_4_	6 g/L	6 g
MgSO_4_ 7H_2_O	2.5 g/L	2.5 g
distilled H_2_O	N/A	1000 mL
**Total**	**N/A**	**1 L**

Store at 4°C for up to 1 year or until precipitates appear.

• lysis buffer (4% [w/v] SDS, 100 mM Tris/HCl pH 8.2, 0.1 M dithiothreitol [DTT]).

Prepare just before use. Avoid light.

• UA buffer (8 M urea in 100 mM Tris/HCl pH 8.2).

Prepare just before use.

• The IAA solution (0.05 M IAA in UA buffer).

Prepare just before use. Avoid light.

• 0.05 M triethyl-ammoniumbicarbonate (TEAB).

Prepare a few weeks in advance and store at 4°C for up to 1 year or until precipitates appear.

## Step-by-step method details

### Primer design and PCR conditions to generate DNA fragments for gene replacement of *hcp* in *A. aphrophilus*

**Timing: 4–5 h**

This section describes the steps to generate DNA fragments for gene replacement of hcp in *A. aphrophilus*.

1. Prepare the DNA template.
  a. Boil a loopful of *A. aphrophilus* colonies (we picked generally 10 colonies) from fresh agar plates in 100 μL Milli-Q ultrapure water for 10 min.
  b. Centrifuge the sample for 1 min at 14,000 rpm using a benchtop Eppendorf centrifuge.
2. For each PCR reaction (final volume 25 μL).
  a. Add 22 μL Milli-Q ultrapure water to Ready-To-Go PCR beads.
    ***Note:*** The reaction used Illustra PuReTaq Ready-To-Go PCR beads and Milli-Q ultrapure water, and was optimized for *A. aphrophilus* strains HK83 and CCUG 11575.
  b. Add 1 μL of each primer (from stock concentrations of 10 μM).
    ***Note:*** Primers were designed using OligoCalc (http://biotools.nubic.northwestern.edu/OligoCalc.html) to ensure compatible melting temperatures and prevent hairpin formation, with BamHI restriction sites added to minimize alterations to the target sequences.
  c. Add 1 μL of the prepared DNA template.
3. Run the PCR with the following settings.
  a. 10 min at 95°C.
  b. 35 cycles of:
    i. 95°C for 30 s.
    ii. 56°C for 30 s.
    iii. 72°C for 1 min.
  c. Followed by 7 min at 72°C.
4. Purify the obtained PCR products, directly after the PCR, using reagents that remove the reaction components. We have used the Qiaquick PCR Purification Kit according to the manufacturer’s instructions.

### Preparation of sTSB agar plates

**Timing: 1–2 h**

Here we outline the preparation of sTSB agar plates, including sterilization and the addition of heat inactivated horse serum, providing a nutrient-rich medium for bacterial growth.

5. For one liter of sTSB agar medium (enough for ≈ 30 plates) suspend 45 g Trypticase soy broth (TSB; Bacto Tryptic Soy Broth, Becton Dickinson, Heidelberg, Germany), 1 g yeast extract, and 15 g agar in 1000 mL of distilled water.
6. Sterilize by autoclaving at 121°C for 15 min.
7. Cool to agar medium to 45°C–50°C.
8. Add aseptically 50 mL of heat inactivated horse serum.
9. Mix and then pour the plates.

### DNA ligation and transformation procedures for *hcp* gene replacement in *A. aphrophilus*

**Timing: 1 week (**s**ee the schematic in our graphical abstract)**

This section describes the steps for DNA digestion, purification, ligation, and bacterial transformation. DNA fragments and plasmid are digested with *Bam*HI, and the kanamycin resistance cassette is purified via gel electrophoresis. The PCR fragments and kanamycin cassette are then ligated, i.e., generating a construct *hcp* upstream – kanamycin – *hcp* downstream, followed by bacterial transformation using *A. aphrophilus*. Finally, transformants are selected for kanamycin resistance and confirmed via PCR.

10. Digestion of DNA Fragments and Plasmid with *Bam*HI:
  a. Prepare separate digestion reactions for PCR fragments and plasmid pUC4K with *Bam*HI. This plasmid is described earlier^14^ and is deposited in GenBank (accession X06404).
  b. Use 500 ng of each DNA sample in a 30 μL reaction mixture.
  c. Incubate the reactions at 37°C for up to 2 h.
11. Purification of Kanamycin (Kmr) Gene Cassette from Plasmid:
  a. Perform agarose gel electrophoresis to separate DNA fragments.
  b. Excise and purify the band containing the 1364 base pair kanamycin resistance cassette using the QIAquick Gel Extraction Kit (Cat. No. 28704, QIAGEN), following the manufacturer’s instructions.
12. Ligation of PCR Fragments and Kanamycin Cassette:
  a. Prepare ligation reactions by mixing equal amounts of PCR fragments (obtained by using the primers *hcp*_F2 plus *hcp*_R2, and *hcp*_F3 plus *hcp*_R3, respectively) flanking *hcp* and the purified kanamycin cassette.
  b. For each ligation reaction, combine the DNA fragments with 1 μL of T4 DNA ligase (Promega) in a total volume of 11 μL.
  c. Achieve a final DNA concentration of approximately 100 μg/mL.
  d. Incubate the ligation reactions at RT overnight.
13. Preparation of Bacterial Cells and Transformation:
  a. Harvest fresh *A. aphrophilus* from cultures grown on sTSB agar plates to a concentration of approximately 5 × 10^9^ CFU/mL in TSB broth.
  b. Spread 20 μL aliquots of bacterial solution on pre-warmed (37°C) blood agar plates in small areas (approximately 10 mm in diameter).
  c. Incubate the plates at 37°C for 2 h.
  d. Mix the bacterial cells on the agar plates with the ligation mixture(s) prepared in step 3.
  e. Incubate the plates at 37°C with 5% CO_2_ for 6 h.
14. Selection and Confirmation of Transformants:
  a. Scrape the bacterial cells from the sTSB agar plates using a loop.
  b. Resuspend the cells in 100 μL of TSB.
  c. Plate the cell suspension onto sTSB agar plates containing 100 μg/mL kanamycin.
  d. Incubate the plates at 37°C for 48 h.
  e. Test the obtained colonies for kanamycin resistance.
  f. Confirm transformants by PCR using the following primers:
    i. The hcp upstream (*hcp*_F2).
    ii. The downstream (*hcp*_R3).
    iii. A primer specific for the kanamycin resistance cassette.
  g. Run the PCR reaction following the PCR cycling conditions as described in step 2 of the PCR protocol.
  h. Confirm the presence of the kanamycin cassette and successful transformation by comparing the sizes of the PCR products to the expected values.

***Note:*** Depending on the orientation of the kanamycin cassette, a PCR product will be obtained with primer H7R either in combination with *hcp*_F2 (≈1,100 base pairs [bp]), or with *hcp*_R3 (≈1,270 bp).

### Dual species agar competition assay

**Timing: 4–5 days**

The protocol below describes the steps for assessing T6SS-dependent killing of *A. actinomycetemcomitans* by *A. aphrophilus*, on agar. Bacterial strains to be used in this protocol need to be cultivated on agar prior to experiments.

15. Preparation of Co-culture and Controls (See the schematic in our graphic abstract):
  a. Harvest *A. aphrophilus* from blood agar plates to achieve OD_600nm_ = 1.7 in Tryptic Soy Broth (TSB).
    ***Note:*** Due to the similar colony morphology of the bacterial strains used in these experiments, spontaneous streptomycin- and rifampicin-resistant derivatives ensure that both species, despite being mixed in the experiments, can be independently enumerated on the blood agar plates containing the respective antibiotic selection. For obtaining spontaneous streptomycin-resistant derivatives of *A. aphrophilus* (strains HK83, and HK83*Δhcp*, and CCUG 11575 and CCUG 11575 *Δhcp*), and rifampicin-resistant derivatives of *A. actinomycetemcomitans* strain D7SS, respectively. Spread aliquots equivalent to approximately (OD_600nm_ ≈ 1.7–2.0) resuspended in TSB onto blood agar plates containing 100 μg/mL of streptomycin or rifampicin. Isolated obtained resistant colonies to use in the following steps. We have confirmed the species identity by PCR, using the PCR beads, and specific oligonucleotide primers targeting *A. actinomycetemcomitans*^12^ and *A. aphrophilus*,^4^ respectively using the PCR cycling conditions described in the original sources for these primers as indicated in the key resources table:
  b. Harvest *A. actinomycetemcomitans* from blood agar plates to achieve OD_600nm_ = 1.3 in TSB.
  c. Maintain monocultures of both strains in TSB medium as controls. We re-streaked them to fresh blood agar plates, which were then placed in the 37°C incubator with 5% CO_2_ until use.
  d. Mix *A. aphrophilus* and *A. actinomycetemcomitans* at a ratio of 3:1 (vol/vol).
  e. Spot 40 μL aliquots of the mixed culture and each monoculture on blood agar plates.
  f. Incubate the plates overnight at 37°C with 5% CO_2_.
16. Harvest and resuspension of bacteria from plates:
  a. Scrape bacteria from the blood agar plates using a loop or sterile swab.
  b. Transfer the scraped material into TSB to resuspend the bacterial cells.
17. Enumeration of colony-forming units (CFU) and Calculation of Killing Index:
  a. Perform serial dilutions of the bacterial suspension.
  b. Spread appropriate dilutions on blood agar plates containing:
    i. Streptomycin (for *A. aphrophilus* selection).
    ii. Rifampicin (for *A. actinomycetemcomitans* selection).
  c. Incubate the plates for two days at 37°C with 5% CO_2_.
  d. Counting CFUs.
  e. After incubation, count the number of CFU for:
    i. *A. aphrophilus* on streptomycin-containing plates.
    ii. *A. actinomycetemcomitans* on rifampicin-containing plates.
  f. Calculate the killing index as described,^1^ i.e., the ratio of *A. aphrophilus* CFU numbers in the co-culture (mixed culture) divided by the CFU numbers when *A. aphrophilus* is in monoculture (alone).

***Note:*** The killing index provides a measure of bacterial competition or antagonism, where a ratio less than 1 indicates potential killing or inhibition executed by the donor strain (*A. aphrophilus*) towards the recipient strain (*A. actinomycetemcomitans*).

### Multi-species biofilm

**Timing: 5 days**

The protocol below describes the steps for developing multi-species biofilm models to mimic the killing activity of *A. aphrophilus* against *A. actinomycetemcomitans* in their natural habitat with other oral species.

18. Day 1:
  a. Prepare two 15 mL tubes for each cell strain with labeled group A and group B.
    ***Note:*** The experiment lasts for 5 days. Thus, we recommended starting the experiment on a Monday while preparing the strains on Friday the week before, as stated in the “before you begin” section.
  b. Aliquot 9 mL FUM with 0.3% Glucose in Sörensen’s buffer (FUM/Sö./0.3%G; please see the materials table below) medium in tubes of group A and 5 mL of the same medium in tubes of group B.
    ***Note:*** The medium used in the experiment is FUM + 0.3% glucose in Sörensen's buffer (FUM/Sö./0.3%G), as described in the “materials and equipment” section above, along with added saliva and human serum. The FUM/Sö./0.3%G medium consists of three parts: Solution A, Solution B, and the Salt Nutrient Buffer (SNB) solution. These components should be prepared separately to avoid precipitation. We recommend preparing Solutions A and B the week before the experiment, while SNB (including SNB1 and SNB2) can be prepared a few weeks in advance and stored at 4°C. The final FUM/Sö./0.3%G medium should be prepared the afternoon before the experiment, with the saliva and human serum added on the same day of the experiment.
  c. Inoculate strains by adding 0.5 mL cell suspensions with OD_550nm_ values around 1 AU (from Friday).
  d. Incubate overnight.
    ***Note:*** Unless stated otherwise, the incubators were aerobic, operating at 37°C with 5% CO₂.
  e. Label 24 well plates for the later experiment.
19. Day 2:
  a. Aliquot 0.5 mL of solutions from group A to ground B and incubate in incubators at 37°C.
  b. Check the turbidity of each bacterial culture, and store those with OD_550nm_ values around 1 AU in a 4°C fridge.
  c. Place 24 hydroxyapatite discs (Clarkson Chromatography Products Inc) into the 24-well plate.
  d. Prepare 20 mL of initial medium (10 mL saliva, 7.5 mL dH2O and 2.5 mL 0.9% NaCl).
  e. Apply 0.8 mL initial medium to each well (with disc).
  f. Leave a 24-well plate on the shaker at 95 rpm for 4 h at room temperature (roughly 20°C–22°C).
  g. Meanwhile, check the turbidity of the strains. Place strains in the 4°C fridge when their OD arrives at around 1.
  h. Prepare 44 mL of growth medium (28 mL saliva, 4 mL human serum and 12 mL FUM/Sö./0.3%G mixture (783.3 μL in 50 mL FUM/Sö./0.3%G medium) and apply 1.6 mL in each well.
  i. Equilibrated 24-well plate in an incubator for at least 45 min before use.
  j. Adjust the OD_550nm_ value to 1 AU (acceptable error range of 0.05 AU).
  k. Check the purity of each medium by microscope.
    ***Note:*** This is a preliminary quality check process. We expect to see only one type of species observed under the microscope (no cross-contamination) and that each species displays its typical morphology. We recommend maintaining a notebook with figures of the typical morphology for all species as a standardized reference for comparison. If any signs of contamination or abnormal morphology are observed, the experiment should be terminated at this stage.
  l. Add 0.5 mL of each different strain to make 5 different mixtures.
     Mixture A: Non-*A. aphrophilus* control.
     Mixture B: All the other bacterial species with *A. aphrophilus* strain HK83.
     Mixture C: All the other bacterial species with *A. aphrophilus* strains CCUG 11575.
     Mixture D: All the other bacterial species with *A. aphrophilus* strain HK83 *Δhcp*.
     Mixture E: All the other bacterial species with *A. aphrophilus* strains CCUG 11575 *Δhcp*.
  m. Transfer discs (after 4 h shaking) to a pre-equilibrated 24-well plate.
  n. Add 200 μL mixture medium into the corresponding well.
  o. Incubate 24-well plates overnight.
20. Day 3 and Day 4:
  a. Make 44 mL of growth medium (28 mL saliva, 4 mL human serum and 12 mL FUM/Sö./0.3%G mixture (783.3 μL in 50 mL FUM/Sö./0.3%G medium) and apply 1.6 mL in each well. Equilibrated in an incubator for at least 45 min before use.
  b. Fill the first row of the wash plate with 1 mL of 0.9% NaCl.
  c. Move hydroxyapatite discs to the first row of the wash plate.
  d. Gently shake plates for 1 min (count after moving the first disc into the wash plate).
  e. Dip each disc 3 times in 3 wells that are filled with 2 mL 0.9% NaCl.
  f. Move dipped discs to a pre-equilibrated medium.
  g. Keep the plate in the incubator for 4 h.
  h. Repeat steps b to d.
  i. Move the dipped discs back into the plate and incubate for another 4 h.
  j. Repeat steps b and d.
  k. Move the dipped discs back into the plate and incubate overnight.
21. Day 5.
  a. Wash discs 3 times in 0.9% NaCl.
  b. Transfer discs into a 50 mL tube (with 1 mL 0.9% NaCl).
  c. Vortex discs 2 min.
  d. Sonicate bacteria for 5 s.
  e. Mix 15 μL bacterial suspensions with 0.3 μL live/dead dye (Invitrogen LIVE/DEAD BacLight Bacterial Viability Kits or other fluorescent staining kits commonly used for assessing the viability of bacterial cells) on cover slides.
  f. Incubate slides in a dark environment for 15 min at room temperature.
  g. Check slides under the fluorescence microscope.
  h. Dilute bacterial suspension into different concentrations.
  i. Plate dilution for CFU counting.

### Protein extraction, digestion, and C18 clean-up

**Timing: 4–5 h, excluding the overnight digestion**

The protocol below describes the steps for extracting proteins from the biofilms before being subjected to the mass spectrometer (MS). In our previously published paper,^1^ we employed reversed-phase HPLC coupled with electrospray ionization (ESI)–MS for our analyses. The HPLC system used comprised a Thermo Scientific EASY-nLC 1200, coupled with a 15 cm-long, 75 μm-diameter silica emitter and ReproSil-Pur C18-AQ 120 Å, 1.9 μm resin (Dr. Maisch HPLC GmbH). We employed a three-step gradient of acetonitrile/water (with 0.1% formic acid) at a flow rate of 300 nL/min: initially, the gradient increased from 2% to 30% acetonitrile over 60 min; followed by an increase from 30% to 97% over 10 min; and finally, maintained at 97% for an additional 10 min. The mass spectrometer, an Orbitrap Fusion (Thermo Fisher Scientific), was operated in data-dependent mode with automatic switching between MS and MS/MS using Xcalibur software (Thermo Fisher Scientific). The Orbitrap analyzer was configured to scan within a mass range of 300–1500 m/z. However, tryptic peptides can also be used in other shotgun proteomics protocols with varying analysis modes, columns, mobile phases, flow rates, and gradients, depending on the specific requirements of the user’s application.

22. Protein extraction from biofilm.
  a. Vortex bacterial suspension, spin down 13,000 rpm for 15 min.
  b. Resuspend pellets with 30 μL lysis buffer.
    ***Note:*** The lysis buffer (4% [w/v] SDS, 100 mM Tris/HCl pH 8.2, 0.1 M dithiothreitol [DTT]) used in the experiment should be prepared on the day of the experiment. Since DTT is sensitive to light, we recommend wrapping them in aluminum foil after preparation.
  c. Incubate at 95°C and 900 rpm for 5 min (at a thermomixer [Eppendorf], or in a water bath without shaking).
    ***Note:*** Use Eppendorf Safe-Lock Tubes to avoid lids popping off or holes on lids (which may cause water to come to the tube during sonification).
  d. Cool the tubes on ice for 40 s.
  e. Resuspend well by pipetting or vox on a bench-top vortex mixer, and spin down the medium to the bottom.
  f. Sonification using 65%–75% amplitude, 0.5 cycles, with floating ice bath for 3 min.
  g. Cool the tubes at room temperature for 3 min.
  h. Repeat the sonication step twice.
  i. Vortex, centrifuge at 14,000 rpm for 20 min.
23. Measure the protein concentrations using Qubit.

***Note:*** A typical biofilm on an 8 mm hydroxyapatite disc will yield 10–30 μg of protein (0.3–1 μg/μL in lysate).

24. Protein digestion.
  a. Aliquot sample with 20 μg protein with 200 μL UA buffer (8 M urea in 100 mM Tris/HCl pH 8.2) and load to the filter unit.
    ***Note:*** The UA buffer (8 M urea in 100 mM Tris/HCl pH 8.2) used in the experiment should be prepared on the day of the experiment.
    ***Note:*** The recommended digestion amount for FASP is 20 μg. However, lower protein weight (down to 10 μg) could be possible, with a risk of reducing proteome coverage or creating outliers for label-free quantification. Analyzing samples with lower protein abundance is not recommended. We suggest first verifying that the biofilm growth is normal. If it is, consider combining multiple biological replicates into a single protein extraction sample.
  b. Sample volumes should be smaller than 30 μl, otherwise divided into different times according to the proportions.
  c. Centrifuge at 14,000 rpm for 20 min at RT or 35°C.
  d. Discard flow-through.
  e. Add 200 μL of UA to the filter unit.
  f. Centrifuge at 14,000 rpm for 20 min at RT or 35°C.
  g. Discard flow-through.
    ***Note:*** No need to change the medium since the collection tube can hold up to 500 μL.
  h. Add 100 μL iodoacetamide (IAA) solution (0.05 M IAA in UA) to a Microcon YM-30 centrifugal filter unit (Millipore).
    ***Note:*** The IAA solution (0.05 M IAA in UA buffer) used in the experiment should be prepared on the day of the experiment. Since IAA is sensitive to light, we recommend wrapping in aluminum foil after preparation. Mix at 600 rpm in the thermo-mixer for 1 min.
  i. Incubate without mixing for 5 min.
  j. Centrifuge at 14,000 rpm for 20 min at RT or 35°C.
  k. Add 100 μL of UA to the filter unit.
  l. Centrifuge at 14,000 rpm for 20 min at RT or 35°C.
  m. Repeat the step two more times.
    ***Note:*** Do not shorten this step – the total volume of wash must be 300 μL.
  n. Add 100 μL of 0.5 M NaCl to the filter unit.
  o. Centrifuge at 14,000 rpm for 17 min at RT or 35°C.
  p. Repeat step one more time – the total volume of wash is 200 μl.
  q. Transfer the filter units to new collection tubes.
  r. Resuspend 0.4 μg trypsin in 120 μL 0.05 M TEAB (a 1:50 ratio of trypsin to the substrate) and load this 120 μL mixture to filter unite.
    ***Note:*** The 0.05 M TEAB solution for trypsin digestion and solutions used for the C18 cleanup method can be prepared a few weeks in advance and stored under cool conditions.
  s. Mix at 600 rpm in the thermo-mixer for 1 min.
  t. Incubate the units overnight on the bench in a wet cell.
  u. Centrifuge the filter units at 14,000 rpm for 17 min to collect the digested peptide.
25. C18 clean up.
  a. Acidify with 5% TFA Solution to the final concentration of 0.5% trifluoroacetic acid (TFA) in new Eppendorf tubes.
  b. Make sure the final solution is acidic (pH 3 or lower) with a universal indicator (MQuant).
  c. Active StageTips (200 μL tip with a C18 disk core (Thermo Scientific)) by load 200 μL mL 100% methanol.
  d. Equilibrate columns by load 200 μL mL 60% acetonitrile (ACN), 0.1% TFA.
  e. Equilibrate columns by load 200 μL 3% ACN, 0.1% TFA.
  f. Load samples on the columns, collect flow through and load it on columns again.
  g. Wash columns by load 1.2 mL 3% ACN, 0.1% TFA.
  h. Elute samples from columns with 200 μL 60% ACN, 0.1% TFA.
  i. Store at −20°C until subject to the mass spectrum.

***Note:*** The peptide samples are now ready for LC-MS/MS analysis. We recommend resuspending the dried peptides with 30 μL of loading buffer (e.g., 3% ACN, 0.1% formic acid) and injecting 2–4 μL of the resuspended sample into the LC-MS/MS. In our paper,^1^ label-free quantitative proteomics identified and quantified 3,286 proteins, with a protein false discovery rate of 0.091%. You can also get more details from our previously published protocol^15^ Quality control (QC) is essential for tracking variations between biofilm samples. However, since this protocol primarily focuses on sample preparation rather than the LC-MS/MS process, QC is not the main emphasis of our paper. We recommend following best practices for sample preparation as outlined earlier,^16^ in accordance with the standards of your lab or local proteomics center. Additionally, we strongly suggest spiking in a known peptide standard (e.g., digested bovine serum albumin) every 4–5 samples to ensure consistent quantification. It is also crucial to ensure that ≥80% of proteins have no missed cleavages (in our study, this was over 90%) to confirm proper tryptic digestion. Furthermore, the coefficient of variation within each biofilm condition should be less than 50% (in our case, it ranged from 10%–30% across five different biofilm conditions).

## Expected outcomes

The protocol evaluates the role of the T6SS in the fitness of the oral symbiont *A. aphrophilus* and its antagonistic effects on the pathobiont *A. actinomycetemcomitans* using a multi-species biofilm model that mimics the oral dental plaque ecosystem. Expected outcomes include the successful design, construction, and validation of T6SS-deficient strains. Additionally, co-culturing *A. aphrophilus* and *A. actinomycetemcomitans* on agar (dual-species agar competition assays) and within the multispecies biofilm (containing these two and five additional organisms on a hydroxyapatite disc) confirms the antagonistic effect of T6SS on *A. actinomycetemcomitans*. This analysis identifies and quantifies proteomic changes in response to T6SS activity, characterizing its influence on microbial interactions. The study provides evidence of T6SS’s active role in shaping the oral microbial community, enhancing the understanding of anti-bacterial strategies employed by oral symbionts. This protocol can also be extended to study other antagonistic effects between oral species in the oral cavity.

For example, variations of biofilms, such as a 10-species 'subgingival' biofilm model (including *Prevotella intermedia*, *Campylobacter rectus*, *V. dispar*, *F. nucleatum*, *S. oralis*, *Treponema denticola*, *Actinomyces oris*, *Streptococcus anginosus*, *Tannerella forsythia*, and *Porphyromonas gingivalis*) or its derivative that includes *A. actinomycetemcomitans*, were also published and could potentially be used for analysis.

## Limitations

The present assays have certain limitations that must be considered. Firstly, although the genomics of the species used in the model is fully annotated, the current database for oral species lacks comprehensive coverage and detail, which can affect the accuracy and completeness of the metaproteomic analysis. This limitation may impact the annotation of quantified bacterial proteins, potentially skewing the characterization of T6SS activity and its effect on microbial interactions within the biofilm. Secondly, while previous studies have shown that it is possible to introduce selected species not part of the normal oral microbiota into an oral biofilm, doing so can dramatically alter the bacterial proportions within the biofilm.^17^ Such changes can affect the dynamics and interactions among the species, potentially leading to misleading results. Therefore, it is crucial to carefully evaluate whether introducing other species is necessary and consider the potential impacts on the composition and behavior of the biofilm before proceeding with such modifications.

## Troubleshooting

### Problem 1

Low reproducibility between multispecies biofilms.

### Potential solution

• Ensure the purity of each strain at the beginning of the experiment (Day 2 step k).
• Ensure consistency in the washing steps for biofilms (Day 3 and Day 4 steps b–e). Prepare extra discs in case of dropping or other accidents during the experiment.
• Remove the “apparent outliers” with very low live bacteria counts (Day 5 step g).
• Vortex the disc thoroughly to ensure proper swirling.

### Problem 2

Protein digestions can be complex, leading to excessive mis-cleavage and generating unusual chromatogram results.

### Potential solution

• Increase the incubation time during protein extraction from the biofilm (step 1) and the sonication duration (steps f–h).
• Always use freshly prepared iodoacetamide.
• Ensure that each centrifugation step completely removes the medium without over-drying the filter during protein digestion.

## Resource availability

### Lead contact

Further information and requests for resources and reagents should be directed to and will be fulfilled by the lead contact, Jan Oscarsson (jan.oscarsson@umu.se).

### Technical contact

Technical questions on executing this protocol should be directed to and will be answered by the technical contact, Kai Bao (kai.bao@ki.se).

### Materials availability

All *A. aphrophilus* strains generated in this study are available upon request.

### Data and code availability

All data reported in this paper will be shared by the lead contact upon request. This paper does not report original code. The mass spectrometry proteomics data have been deposited to the ProteomeXchange Consortium via the PRIDE^18^ partner repository with the dataset identifier PXD042723. Any additional information required to reanalyze the data reported in this paper is available from the lead contact upon request.

## Acknowledgments

This work was funded by grants from the Swedish Research Council (2017-01198 and 2021-03528, to N.B.), strategic funds from Karolinska Institutet (to G.N.B. and N.B.), and TUA grants from Region Västerbotten, Sweden (7002667, to J.O.), and by funds from Insamlingsstiftelsen, Medical Faculty, Umeå University (to J.O.). We would like to thank Dr. Marek Basler and Dr. Karina Persson for valuable discussions and Manuela Flury, Elisabeth Granström, and Carina Öhman for their excellent technical advice and assistance.

## Author contributions

All authors have made substantial contributions to the conception and design of the study. J.O., K.B., G.N.B., and N.B. were responsible for the study concept and design. J.O., K.B., A.S., J.G., W.W., K.M.A., M.L., A.J., F.R.M., and N.B. have been involved in data collection and data analysis. All authors have been involved in data interpretation, drafting the manuscript, and revising it critically and have given final approval of the version to be published.

## Declaration of interests

The authors declare no competing interests.
